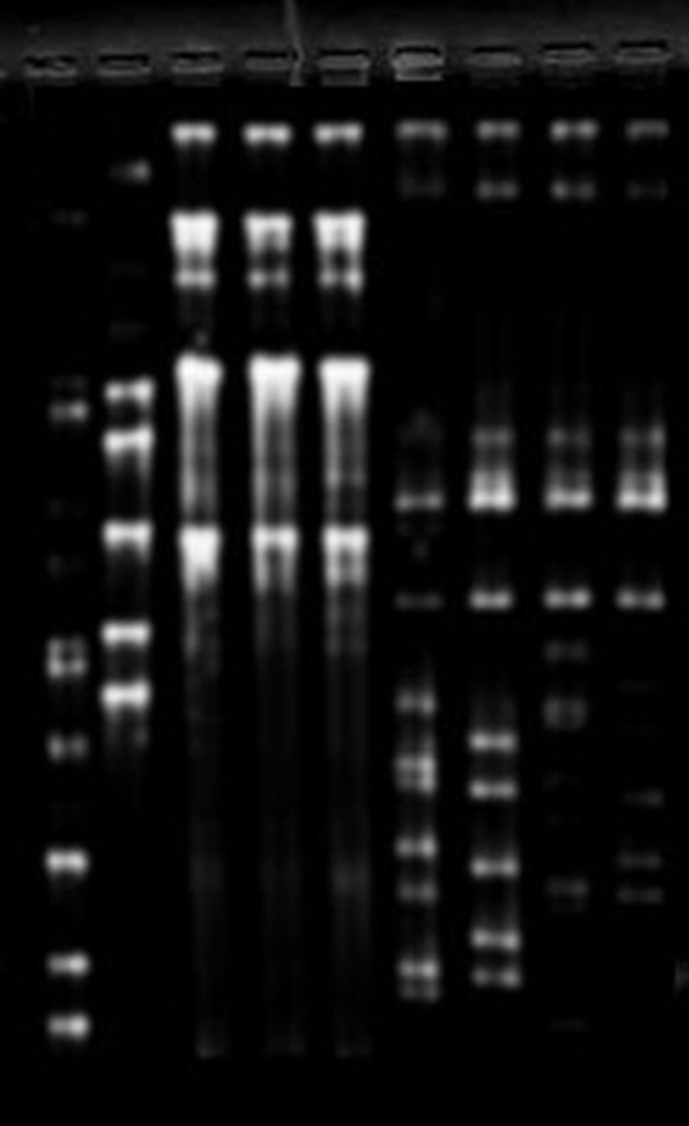# Amelioration of mercuric chloride-induced physiologic and histopathologic alterations in rats using vitamin E and zinc chloride supplement

**DOI:** 10.1016/j.heliyon.2022.e12036

**Published:** 2022-12-05

**Authors:** Mohamed Gaber Shalan

**Affiliations:** Zoology Department, Faculty of Science, Arish University, North Sinai, Egypt

**Keywords:** Mercuric chloride, Vitamin E, Zinc chloride, Oxidative stress, Apoptosis

## Abstract

The drastic effects of mercuric chloride and the protective efficiency of vitamin E and zinc chloride co-supplementation were clearly investigated in this study. Male rats were divided into four groups. The first was the control. The second received vitamin E (100 mg/kg) and zinc chloride (30 mg/kg) daily. In comparison, the third received mercuric chloride (1 mg/kg) daily, and the fourth received the same mercuric chloride dose supplemented with the same vitamin E and zinc chloride doses. Mercury promotes a significant decline in body weight. It causes a considerable reduction in total red blood cells (RBCs) count and hemoglobin concentration; however, white blood cells (WBCs) increased significantly. Significant mercury-induced elevations in hepatic and renal functions were observed. Mercury induced substantial reductions in catalase (CAT) and superoxide dismutase (SOD). Mercury caused apoptotic DNA fragmentation. It induced degeneration and necrosis in the liver and kidney. It induced necrosis, leukocyte infiltration and blood vessel congestion in the cerebral cortex. Shrinkage and deterioration of Purkinje cells of the cerebellum were observed in response to mercuric chloride toxicity. Mercuric chloride enhanced shrinking in seminiferous tubules and Leydig cells. It reduced sperm count, sperm motility, and testosterone concentration; however, it promoted abnormal sperm morphology. Administration of vitamin E and zinc chloride showed marked improvement in different parameters under investigation, however, further research is needed to determine fate of mercury.

## Highlights

• Mercuric chloride is a potentially hazardous toxin and is highly spread.
• Mercury exposure induced lipid peroxidation and oxidative stress.
• Mercuric chloride induces histopathological and physiological alterations in different body organs.
• Combined vitamin E and zinc chloride supplementation reduce mercuric chloride toxicity.

## 1 Introduction

Mercury is one of the most serious environmental pollutants (Mesquita et al., 2016; Nabil et al., 2020), which can be found in water, air and soil. There are several sources of mercury exposure, including dental amalgam fillings (Bengtsson and Hylander, 2017), seafood (Dadar et al., 2016; Kuras et al., 2017), vaccines (Mitkus et al., 2014) and energy-saving light bulbs (Bjorklund et al., 2017). The most toxic form of mercury is mercuric chloride because of its affinity for proteins (Boujbiha et al., 2009). Mercury was found to affect the liver (Uzunhisarcikli et al., 2015), kidney (Aslanturk et al., 2014), brain (Franco et al., 2007) and testis (Kalender et al., 2013). Mercury is metabolized in the liver and accumulated in body organs (Bridges and Zalups, 2017). Mercury enhances lipid peroxidation and oxidative stress (Salman et al., 2016). The formed free radicals cause deterioration in body organs (Cappelletti et al., 2019).

Many trials have been done for attenuating mercuric chloride toxicity using vitamin E (Al-Attar, 2011), vitamin E in combination with vitamin C (Muthu and Krishnamoorthy, 2012), sodium selenite (Celikoglu et al., 2015), Lactobacillus (Alhusaini et al., 2021). Other studies include zinc alone (Fukino et al., 2009; Oliveira et al., 2014), Scorpion venom alone (Salman et al., 2016) and others (Cappelletti et al., 2019; Nabil et al., 2020). None of these materials prove complete protection against mercury toxicity. Vitamin E is a potent chain braking antioxidant (Celikoglu et al., 2015), and zinc chloride activates many enzymes (Fukino et al., 2009; Oliveira et al., 2014). Combined vitamin E and zinc chloride supplementation for ameliorating mercuric chloride's toxic effects has received little attention. The objective of this work was to demonstrate the damage induced by mercury in four experimental time intervals, in addition to understanding the protective role of vitamin E and zinc chloride supplementation against mercuric chloride toxicity.

## 2 Materials and methods

### 2.1 Chemicals

Mercuric chloride (HgCl_2_, 99% purity), Vitamin E (DL-α-tocopherol acetate) that is dissolved in olive oil and zinc chloride (ZnCl_2_) were purchased from Sigma Aldrich, Germany.

### 2.2 Animals

Eighty (80) male albino rats (*Rattus norvigicus*), purchased from the Egyptian Organization for Biological and Vaccine Production, Arab Republic of Egypt, seven weeks old, weighing 90 ± 10g, were used as experimental animals. Animals were housed in 4 groups in plastic cages throughout the present study.

Animals were maintained on a 12 h light/dark cycle and at a controlled temperature (25±2 °C) and relative humidity (45 ± 5%). Animals were fed a standard balanced diet and water *ad libitum* and acclimatized for ten days to the lab conditions. The Research Ethics Committee, Faculty of Science, Suez Canal University, approved the experimental protocol for the Ethics of Animal Experimentation.

### 2.3 Experimental design

Animals were randomly subdivided into four groups, each consisting of 20 rats. The first was normal controls. The second received 100 mg/kg of vitamin E (Abd El Fadil et al., 2020) and 30 mg/kg of zinc chloride (V.E + ZnCl_2_ group) (Moazedi et al., 2007) by gastric tube daily. The third received 1 mg/kg mercuric chloride (HgCl_2_ group) daily by gastric tube (Heath et al., 2012). The fourth received 100 mg/kg vitamin E and 30 mg/kg zinc chloride, and 1 mg/kg mercuric chloride (V.E + ZnCl_2_+HgCl_2_ group) by gastric tube daily.

### 2.4 Duration time

Animals were killed by pithing, then rapidly dissected and tissues processed. After 1,2,3 and 4 weeks of treatment, all changes were compared with values recorded in control rats.

### 2.5 Collection of plasma samples

Blood samples were collected from the abdominal vein in EDTA tubes, then centrifuged for 15 min at 1000*g* and stored at -30 °C for further biochemical analysis.

### 2.6 Hematological indices

Hematological measurements (RBCs, Hg & WBCs) were done using a complete blood cell count DIAGON Ltd-D-Cell 60 fully automatic hematological analyzer.

### 2.7 Preparation of tissues for microscopical and gel examinations

After cranial and spinal pithing of rats, liver, brain, kidney and testis were taken, blotted on filter paper and weighed. Each organ is divided into three parts; the first part was kept in a formal saline solution for histochemical studies. The second part was taken immediately for gel and testicular examinations and the remaining portion was stored at -30 °C.

### 2.8 Histopathological methods

Five-micron thick histological sections were prepared and stained with hematoxylin and eosin. Microscopic analysis of the specimens was done blindly (Edrissi, 2022).

### 2.9 DNA extraction

Tissue homogenates (200 mg) were subjected to DNA extraction using the Zymoresearch Quick-g DNA™ MiniPrep kit, Catalog No. D3024, USA. Tissue homogenates were centrifuged at 12,000*g* for 10 min at four °C. Residual supernatants were used for DNA isolation (Naccache et al., 2013).

### 2.10 Detection of the extracted DNA using agarose gel electrophoresis

The DNA product was detected using agarose gel electrophoresis at the end of the extraction process.

**Reagents:**

Agarose (Ultra-pure agarose, electrophoresis grade)

a) Tris-Acetate EDTA buffer (TAE) was prepared by adding 20 ml of 50x TAE buffer to 980 ml distilled water. The 50 x TAE buffer contains:
121 g Tris base, 28.55 ml glacial acetic acid, 50 ml of 0.5 EDTA, pH 8, and completed to 500 ml with distilled water.
b) Ethidium Bromide (EB): This stain was prepared as a stock solution of 10 mg/ml by adding 10 mg of EB powder to 1 ml distilled water and mixing well by the vortex. EB was stored in a dark tube wrapped in aluminum foil at approximately 4 °C. This dye was incorporated into the gel at 0.5 μg/ml concentration.
c) A gel control DNA marker was used in the test.
d) Gel loading buffer (10 ml)

**Equipment:**

a) Hybrid horizontal gel tank: (Biometra).
b) UV transilluminator.
c) Gel Documentation System. Biodoc. Analyzer. (Biometra).

**Procedure:**

a) 2% Agarose gel was prepared as follows: one gram of agarose was dissolved in 50 ml of TAE buffer in a flask covered with aluminum foil for 5 min in a microwave adjustable to medium temperature.
b) When the agarose solution cooled to 60 °C, 2.5 μl of EB was added to allow subsequent visualization of the DNA Gloves were used when working with solutions containing EB dye because it is a potent mutagen and is moderately toxic.
c) The gel was poured into a clean and dry gel mold. When the agarose had set completely (20–30 min), the comb was carefully removed and the gel was placed in the electrophoresis chamber. The sample wells are positioned at the gel tank's opposing electrode end, so DNA migrates through the gel toward the positive electrode. TAE buffer was added just enough to cover the gel.
d) The lid of the electrophoresis chamber was closed, and the gel ran at 100 V, 250 A for 20 min.
e) The DNA was visualized by placing the gel on a UV transilluminator using a 100 bp DNA ladder (Jena Bioscience, Germany) as recorded by the gel documentation system (Lee et al., 2012).

### 2.11 Biochemical analysis

Parameters indicating oxidative stress were measured using Biodiagnostic kits, Egypt. Superoxide dismutase (SOD) activity was measured according to Nishikimi et al. (1972). Catalase (CAT) activity was measured by Aebi (1984) method. Malondialdehyde (MDA) concentration was determined using the technique of Ohkawa et al. (1979).

Glutamic pyruvic transaminase (GPT) and Glutamic oxaloacetic transaminase (GOT) activities were measured by the modified method of Schumann and Klauke (2003).

Uric acid was measured using Spinreact kits, Spain, using the method of Fossati et al. (1980). Creatinine was quantified using Diamond Diagnostics kits, Holliston, the USA, by Heinegaard and Tiderstrom (1973) method. Mercury concentration was measured in whole blood, urine, liver and kidney homogenates using cold vapor atomic absorption spectrometry (Kopp et al., 1972).

### 2.12 Testicular indices

Plasma testosterone was determined using a sandwich immunoassay of iFlash kits, UK, using the method of Wheeler (1995). Sperms count and sperm motility were measured according to Dominic and Padmaja (2017). However, abnormal sperm morphology was detected by Ekaluo et al. (2009).

### 2.13 Statistical analysis

The results represent the means ± standard deviation (SD) for five rats in each group. Data of control and different time intervals of treated rats were examined by one-way analysis of variance (ANOVA). The Tukey test examined multiple comparisons. Difference was considered significant when p˂0.05. Statistical Package for Social Sciences (SPSS) software for windows version 22.0 was used in performing statistical analysis.

## 3 Results

### 3.1 Weights

**Figure 1:**
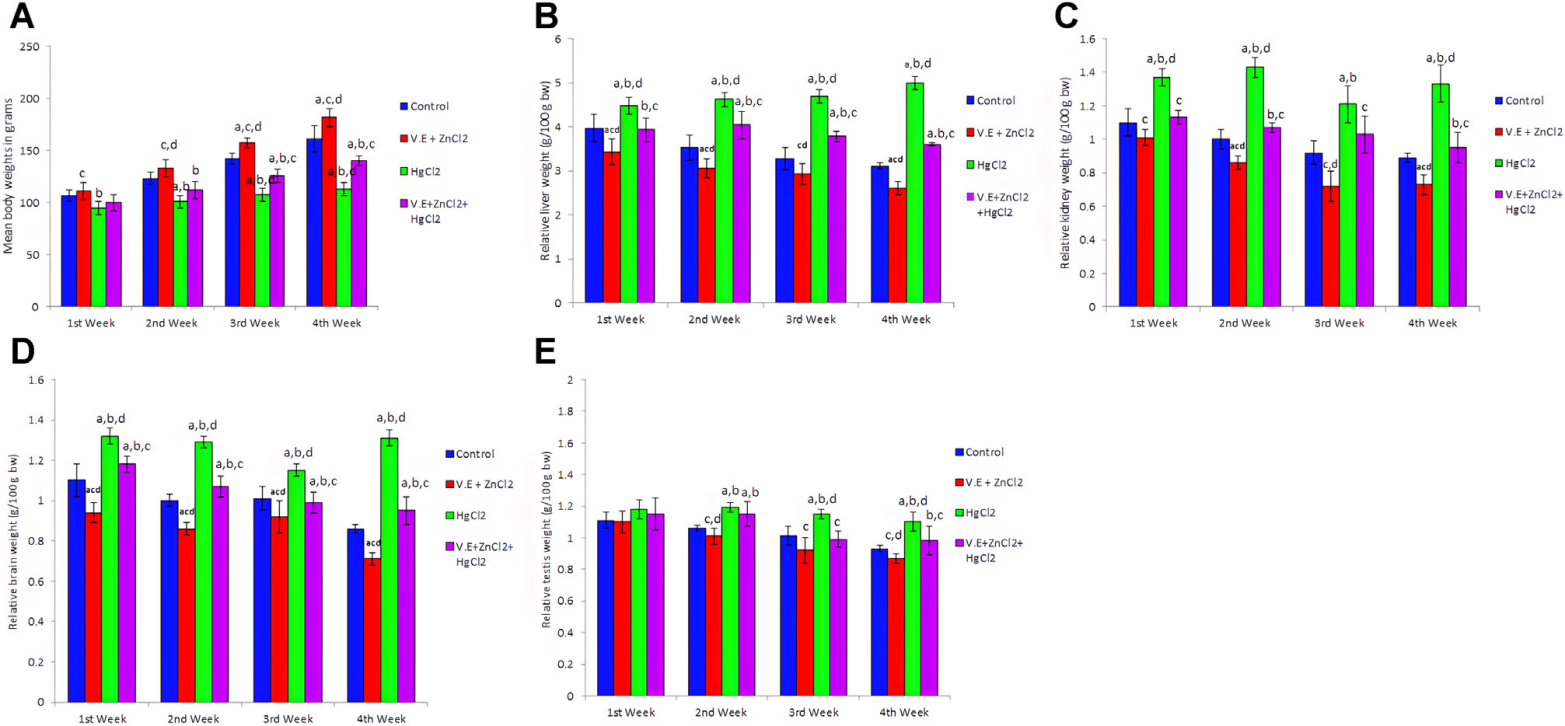
Effects of exposure to mercuric chloride on body weight (A), relative liver weight (g/100 g bw) (B), relative kidney weight (g/100 g bw) (C), relative brain weight (g/100 g bw) (D) and relative testis weight (g/100 g bw) (E) of rats. HgCl_2_: mercuric chloride; V.E: vitamin E; ZnCl_2:_ zinc chloride; bw: body weight. Data are presented as mean ± SD for five rats in each group. a: Significantly different than control group at *p < 0.05* (Tukey's post hoc test), b: Significantly different than vitamin E and zinc chloride group at *p < 0.05* (Tukey's post hoc test), c: Significantly different than mercuric chloride group at *p < 0.05* (Tukey's post hoc test), d: Significantly different than mercuric chloride + vitamin E + zinc chloride group at *p < 0.05* (Tukey's post hoc test).

Treatment with mercury for four weeks induced a significant (*p < 0.05*) decrease in body weight (Figure 1A). However, relative liver, kidney, brain and testis weights were increased significantly in response to chronic mercury toxicity (Figure 1B-E). Supplementation with vitamin E and zinc chloride improved these effects (Figure 1A-E).

### 3.2 Hematology

**Figure 2:**
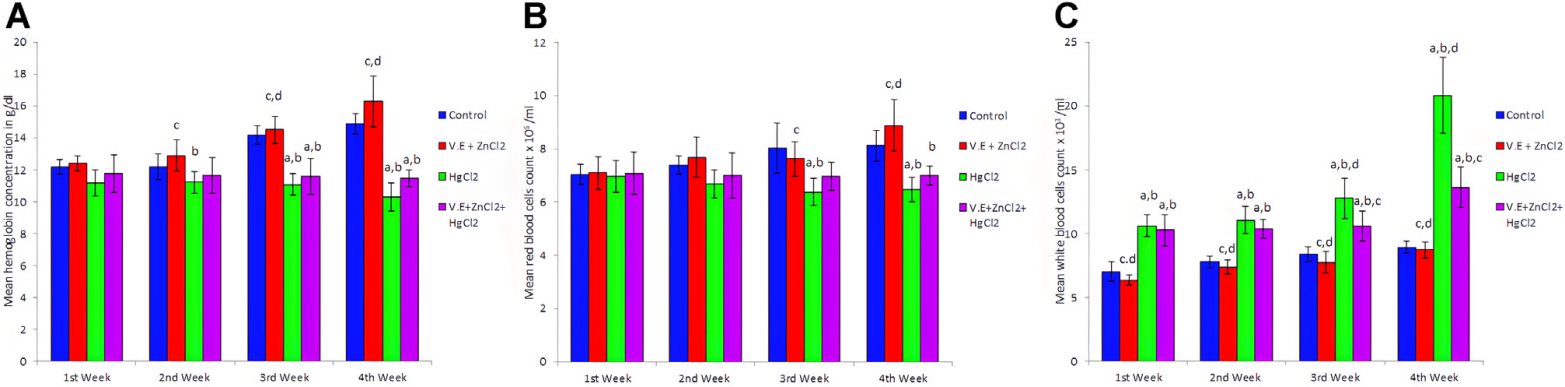
Effects of mercuric chloride on hemoglobin (A), red blood cells (B) and white blood cells (C). HgCl_2_: mercuric chloride; V.E: vitamin E; ZnCl_2:_ zinc chloride. Data are presented as mean ± SD for five rats in each group. a: Significantly different than control group at *p < 0.05* (Tukey's post hoc test), b: Significantly different than vitamin E and zinc chloride group at *p < 0.05* (Tukey's post hoc test), c: Significantly different than mercuric chloride group at *p < 0.05* (Tukey's post hoc test), d: Significantly different than mercuric chloride + vitamin E + zinc chloride group at *p < 0.05* (Tukey's post hoc test).

Chronic mercury toxicity reduced hemoglobin (Hb) concentration in blood significantly (p < 0.05) by 21.86, and 30.69% after 3 and 4 weeks of treatment, respectively (Figure 2A). The red blood cells (RBCs) counts decreased significantly (p < 0.05) by 20.47, and 27.25% of normal controls after 3 and 4 weeks of treatment, respectively (Figure 2B). Total White blood cells increased significantly (p < 0.05) under mercury toxicity by 51.71, 42.16,52.63 and 133.2% after 1,2,3 and 4 weeks of treatment respectively (Figure 2C). Supplementation with vitamin E and zinc chloride improve these effects.

### 3.3 Biochemistry

**Figure 3:**
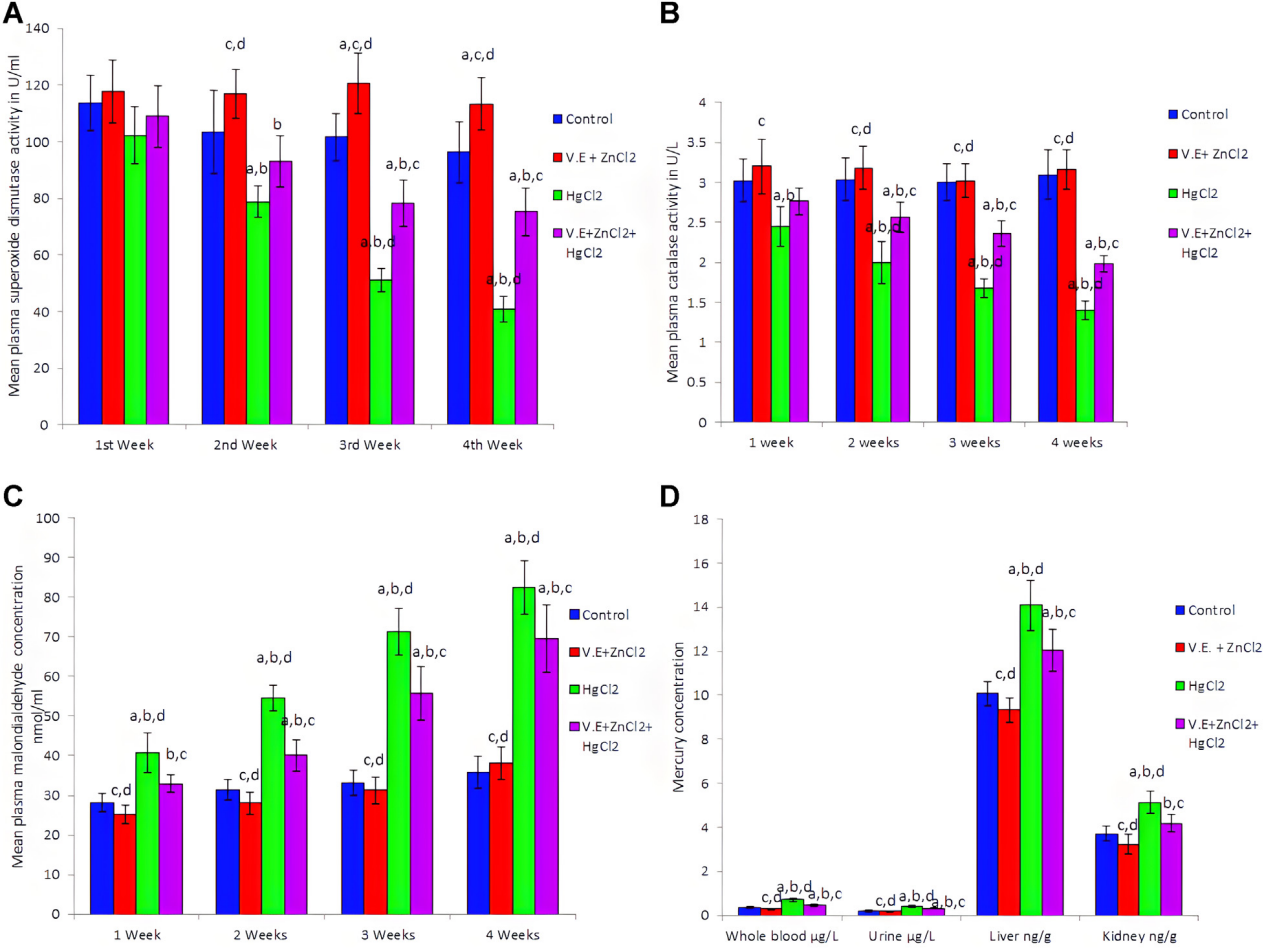
Effects of mercuric chloride on plasma SOD. (A), CAT (B), activities, MDA (C) and mercury (D) concentrations. HgCl_2_: mercuric chloride; V.E: vitamin E; ZnCl_2:_ zinc chloride. Data are presented as mean ± SD for five rats in each group. a: Significantly different than control group at *p < 0.05* (Tukey's post hoc test), b: Significantly different than vitamin E and zinc chloride group at *p < 0.05* (Tukey's post hoc test), c: Significantly different than mercuric chloride group at *p < 0.05* (Tukey's post hoc test), d: Significantly different than mercuric chloride + vitamin E + zinc chloride group at *p < 0.05* (Tukey's post hoc test).

The Consumption of mercuric chloride led to a significant (p < 0.05) decrease in the activity of plasma superoxide dismutase by 23.69, 49.67 and 57.48% of normal controls after 2,3 and 4 weeks of treatment (Figure 3A). At the same time, plasma catalase activity decreased significantly (*P < 0.05*) by 18.87, 33.99, 44.33 and 54.37% compared with normal control rats after 1,2,3 and 4 weeks of treatment respectively (Figure 3B). On the other hand plasma malondialdehyde concentrations increased significantly (*P < 0.05*) amounted to 45.3, 74.25, 115.98 and 129.78% of normal controls in response to the toxicity of mercuric chloride over 1,2,3 and 4 weeks of treatment (Figure 3C).

Mercury concentrations elevated significantly (*P < 0.05*) in whole blood, urine, liver and kidney homogenates after four weeks of mercury exposure (Figure 3D). Plasma GPT, GOT, uric acid and creatinine levels were increased significantly (*P < 0.05*), particularly after chronic mercury toxicity (Table 1).

**Table 1:** Effects of mercuric chloride on liver and kidney function tests and the protective effect of vitamin E and zinc chloride supplementation in male albino rats.

	GPT (U/L)	GOT (U/L)	Uric acid (mg/dl)	Creatinine (mg/dl)
1^st^ week				
Control	28.6 ± 3.05	89.8 ± 7.95	1.63 ± 0.142	0.57 ± 0.070
V.E + ZnCl_2_	26.0 ± 1.58^c,d^	81.4 ± 9.07^c,d^	1.48 ± 0.147	0.53 ± 0.062^c^
HgCl_2_	40.4 ± 4.61^a,b,d^	119.2 ± 11.32^a,b^	1.76 ± 0.186	0.70 ± 0.086^a,b^
V.E + ZnCl_2_ + HgCl_2_	33.4 ± 3.36^b,c^	114.6 ± 9.71^a,b^	1.65 ± 0.158	0.64 ± 0.074
2^nd^ week				
Control	26.6 ± 2.7	92.40 ± 8.17	1.70 ± 0.148	0.60 ± 0.051
V.E + ZnCl_2_	25.2 ± 2.78^c,d^	84.60 ± 9.24^c,d^	1.66 ± 0.169^c^	0.57 ± 0.065^c,d^
HgCl_2_	44.0 ± 4,47^a,b,d^	156.6 ± 16.74^a,b^	2.05 ± 0.242^a,b^	0.92 ± 0.103^a,b,d^
V.E + ZnCl2 + HgCl_2_	34.4 ± 4.28^a,b,c^	134.2 ± 14.33^a,b^	1.78 ± 0.200	0.74 ± 0.081^b,c^
3^rd^ week				
Control	26.8 ± 3.19	111.8 ± 9.28	2.03 ± 0.225	0.58 ± 0.103
V.E + ZnCl_2_	26.4 ± 3.05^c,d^	89.80 ± 8.84^c,d^	1.92 ± 0.150^c^	0.57 ± 0.088^c^
HgCl_2_	49.4 ± 4,39^a,b^	194.2 ± 19.69^a,b,d^	2.62 ± 0.195^a,b,d^	1.06 ± 0.119^a,b^
V.E + Zn Cl_2_ + HgCl_2_	43.2 ± 4.15^a,b^	168.8 ± 10.94^a,b,c^	2.09 ± 0.257^c^	0.90 ± 0.081
4^th^ week				
Control	26.0 ± 3.16	119.8 ± 9.78	2.15 ± 0.210	0.62 ± 0.106
V.E + ZnCl_2_	24.4 ± 3.36^c,d^	99.20 ± 9.96^c,d^	1.96 ± 0.208^c^	0.61 ± 0.105^c,d^
HgCl_2_	54.4 ± 4.98^a,b,d^	217.2 ± 18.30^a,b,d^	3.06 ± 0.383^a,b,d^	1.38 ± 0.176^a,b,d^
V.E + ZnCl_2_ + HgCl_2_	39.2 ± 4.32^a,b,c^	173.0 ± 21.51^a,b,c^	2.42 ± 0.278^a,b,c^	0.98 ± 0.094^a,b,c^

Data are presented as mean ± SD (n = 5). a: Significantly different than normal control group at *p < 0.05* (Tukey's post hoc test), b: Significantly different than vitamin E and zinc chloride group at *p < 0.05* (Tukey's post hoc test), c: Significantly different than mercuric chloride group at *p < 0.05* (Tukey's post hoc test), d: Significantly different than mercuric chloride + vitamin E + zinc chloride group at *p < 0.05* (Tukey's post hoc test).

Mercury induced significant (*P < 0.05*) depletion in testosterone, sperm count and sperm motility, especially with chronic exposure (Table 2). On the other hand, mercury was found to increase abnormal sperm morphology percent significantly (*P < 0.05*) with magnitudes of 33.33, 100, 183.3 and 316.66% after 1,2,3 and 4 weeks of mercury exposure, respectively (Table 2). Supplementation with Vitamin E and ZnCl_2_ refine the biochemical alterations induced by mercuric chloride.

**Table 2:** Effects of mercuric chloride on plasma testosterone, sperm parameters and the protective effect of vitamin E and zinc chloride supplementation in male albino rats.

	Testosterone (ng/dl)	Sperm count x 10^6^/ml	Sperm motility (%)	Abnormal sperm morphology (%)
1^st^ week				
Control	28.3 ± 2.60	102.6 ± 8.05	90.2 ± 6.79	4.80 ± 0.84
V.E + ZnCl_2_	30.8 ± 2.94^c^	111.9 ± 12.3^c^	91.6 ± 6.11	2.80 ± 0.84^a,c^
HgCl_2_	25.8 ± 1.85^b^	93.24 ± 11.2^b^	84.6 ± 6.03	6.40 ± 1.14^b,d^
V.E + ZnCl_2_ + HgCl_2_	28.8 ± 2.17	101.1 ± 8.94	87.6 ± 8.33	3.40 ± 1.14^c^
2^nd^ week				
Control	30.1 ± 2.52	109.6 ± 11.0	92.4 ± 4.39	5.00 ± 1.23
V.E + ZnCl_2_	32.5 ± 2.96^c,d^	115.8 ± 11.4^c,d^	95.6 ± 5.64^c^	2.80 ± 0.84^c,d^
HgCl_2_	20.5 ± 2.34^a,b,d^	82.88 ± 6.78^a,b^	80.6 ± 8.91^b^	10.0 ± 1.58^a,b,d^
V.E + ZnCl_2_ + HgCl_2_	26.4 ± 2.50^b,c^	95.84 ± 9.84^b^	84.8 ± 9.58	6.00 ± 1.58^b,c^
3^rd^ week				
Control	31.0 ± 3.03	120.3 ± 9.43	92.8 ± 7.98	4.80 ± 0.84
V.E + ZnCl_2_	32.8 ± 3.46^c,d^	120.6 ± 9.76^c,d^	94.6 ± 8.29^c^	2.80 ± 0.84^a,c,d^
HgCl_2_	16.4 ± 1.60^a,b,d^	71.50 ± 8.10^a,b,d^	72.6 ± 6.95^a,b^	13.6 ± 1.14^a,b,d^
V.E + ZnCl_2_ + HgCl_2_	25.5 ± 2.51^a,b,c^	90.34 ± 8.94^a,b,c^	82.4 ± 6.95	9.00 ± 1.00^a,b,c^
4^th^ week				
Control	32.6 ± 3.79	118.5 ± 9.40	92.6 ± 4.93	4.40 ± 0.106
V.E + ZnCl_2_	34.4 ± 3.78^c,d^	127.4 ± 6.78^c,d^	95.4 ± 6.43^c,d^	2.40 ± 0.55^c,d^
HgCl_2_	14.8 ± 1.18^a,b,d^	58.90 ± 8.24^a,b,d^	58.0 ± 5.70^a,b,d^	21.8 ± 2.86^a,b,d^
V.E + ZnCl_2_ + HgCl_2_	22.6 ± 2.05^a,b,c^	81.86 ± 5.01^a,b,c^	80.0 ± 3.54^a,b,c^	10.0 ± 1.58^a,b,c^

Data are presented as mean ± SD (n = 5). a: Significantly different than normal control group at *p < 0.05* (Tukey's post hoc test), b: Significantly different than vitamin E and zinc chloride group at *p < 0.05* (Tukey's post hoc test), c: Significantly different than mercuric chloride group at *p < 0.05* (Tukey's post hoc test), d: Significantly different than mercuric chloride + vitamin E + zinc chloride group at *p < 0.05* (Tukey's post hoc test).

### 3.4 Molecular biology

**Figure 4:**
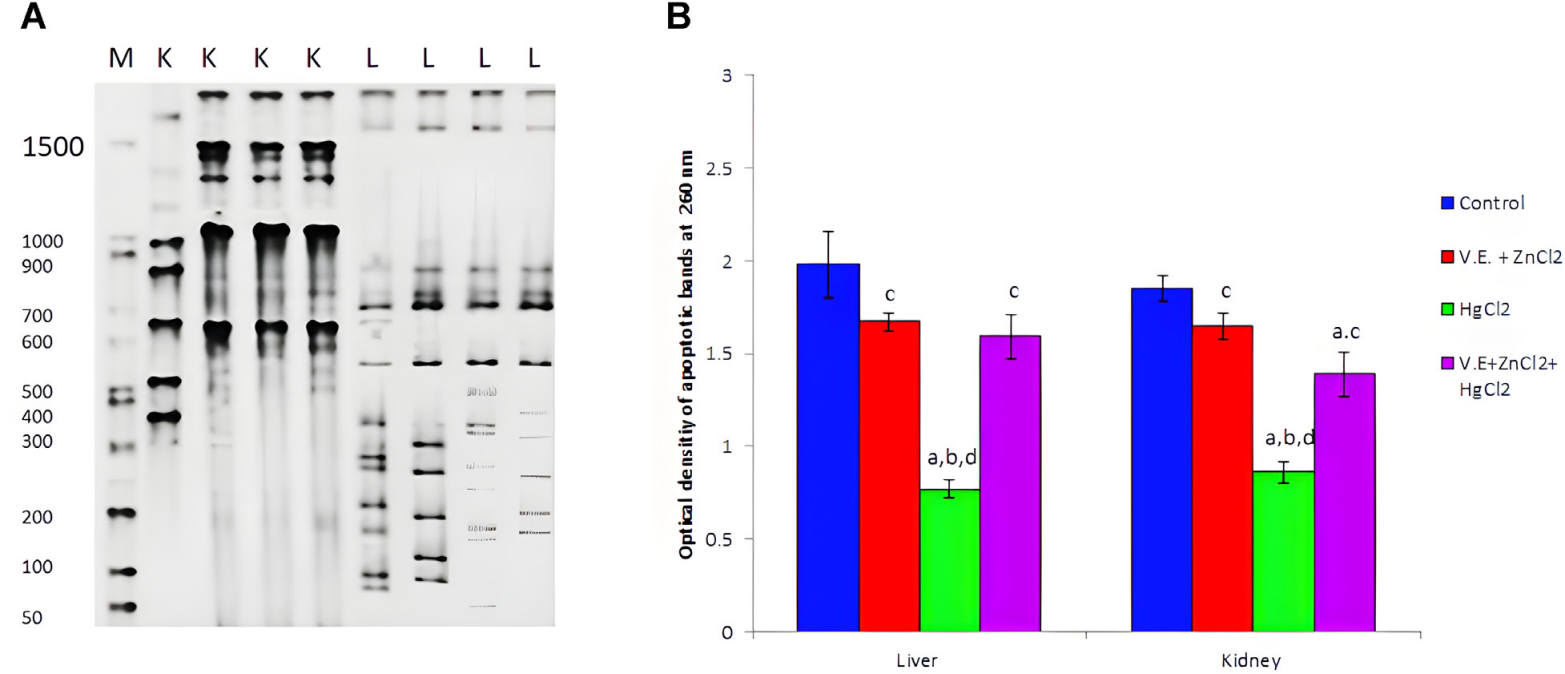
Apoptotic DNA fragmentation in liver (L) and Kidney (K) of rats after 4weeks of mercuric chloride treatment (A), *"from left to right"* Lane 1 (M): 1 Kp ladder; lane 2 (K) control group; lane 3 (K) Vitamin E + zinc chloride group, Lane 4 (K) mercuric chloride group; lane 5 (K) Vitamin E + zinc chloride + mercuric chloride group; lane 6 (L) control group; lane 7 (L) Vitamin E + zinc chloride group, Lane 8 (L) mercuric chloride group; lane 9 (L) Vitamin E + zinc chloride + mercuric chloride group. Optical density of apoptotic bands (B) at 260 nm in liver (L) and kidney (K) of rats after four weeks of mercuric chloride exposure. Data are presented as mean ± SD for five rats in each group. a: Significantly different than the normal control group at *p < 0.05* (Tukey's post hoc test), b: Significantly different than vitamin E and zinc chloride group at *p < 0.05* (Tukey's post hoc test), c: Significantly different than mercuric chloride group at *p < 0.05* (Tukey's post hoc test), d: Significantly different than mercuric chloride + vitamin E + zinc chloride group at *p < 0.05* (Tukey's post hoc test). Non-adjusted image of (4A) was enclosed as supplement file.

As shown in figure (4A, B), chronic mercury ingestion for four weeks induced DNA fragmentation in rats' liver and kidney tissues. However, providing vitamin E and zinc chloride amended mercury toxicity and was influential in restoring DNA.

### 3.5 Histopathological findings

**Figure 5:**
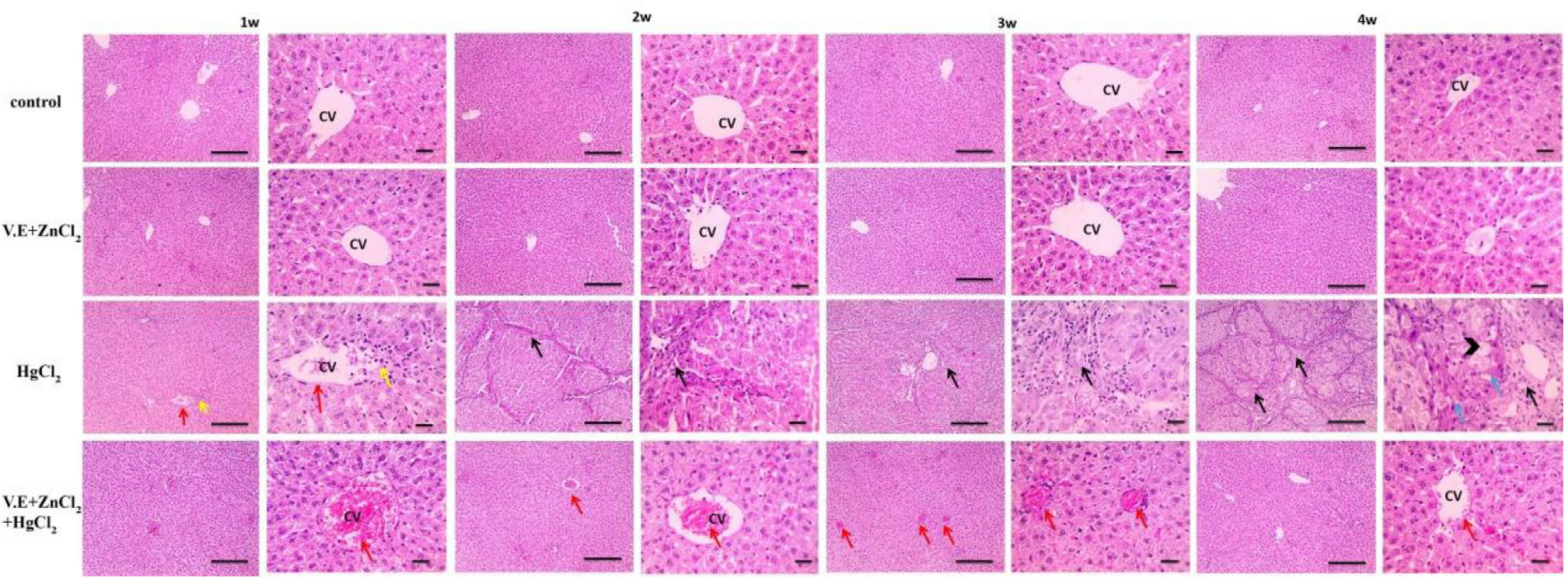
Microscopic pictures of Hematoxylin & Eosin stained liver sections showing normal hepatocytes arranged in radiating plates around the central vein (CV) with normal portal areas and sinusoids in the control group and group receiving zinc chloride and vitamin E. Meanwhile, liver sections from the mercuric chloride group showed mildly congested blood vessels (red arrows) with few perivascular leukocytic cell infiltration (yellow arrow) after one week. Portal areas were expanded due to fibrous tissue proliferation, leukocytic cell infiltration (black arrows) forming elongated strands (black arrows) invading and distorting hepatic parenchyma after two weeks. These lesions increased in severity with time after three weeks and four weeks, where extensive fibrosis (black arrows) appears, dividing hepatic parenchyma into lobules accompanied by hydropic degeneration (blue arrows) and necrosis (black arrowheads) of hepatocytes. Liver sections from the mercuric chloride + vitamin E + zinc chloride group show congested blood vessels (red arrows) with normally arranged hepatocytes around the central vein (CV). The severity of congestion gradually decreases from 2 weeks to 4 weeks, where the liver sections retained normal histological pictures of hepatocytes, central vein (CV), portal areas and sinusoids. Low magnification X: 100 bar 100, high magnification X: 400 bar 50.

Both control and vitamin E, zinc chloride supplemented groups displayed the typical histological architecture of hepatocytes, portal area and sinusoids. After one week of mercuric chloride ingestion, liver tissue showed mildly congested blood vessels with perivascular leukocyte infiltration. Then after two weeks, portal areas were expanded due to the propagation of fibrous tissue. These lesions increased after 3 and 4 weeks of mercury toxicity, with extensive fibrosis, degeneration and necrosis of hepatocytes (Figure 5). Administration of vitamin E and zinc chloride refined these effects.

**Figure 6:**
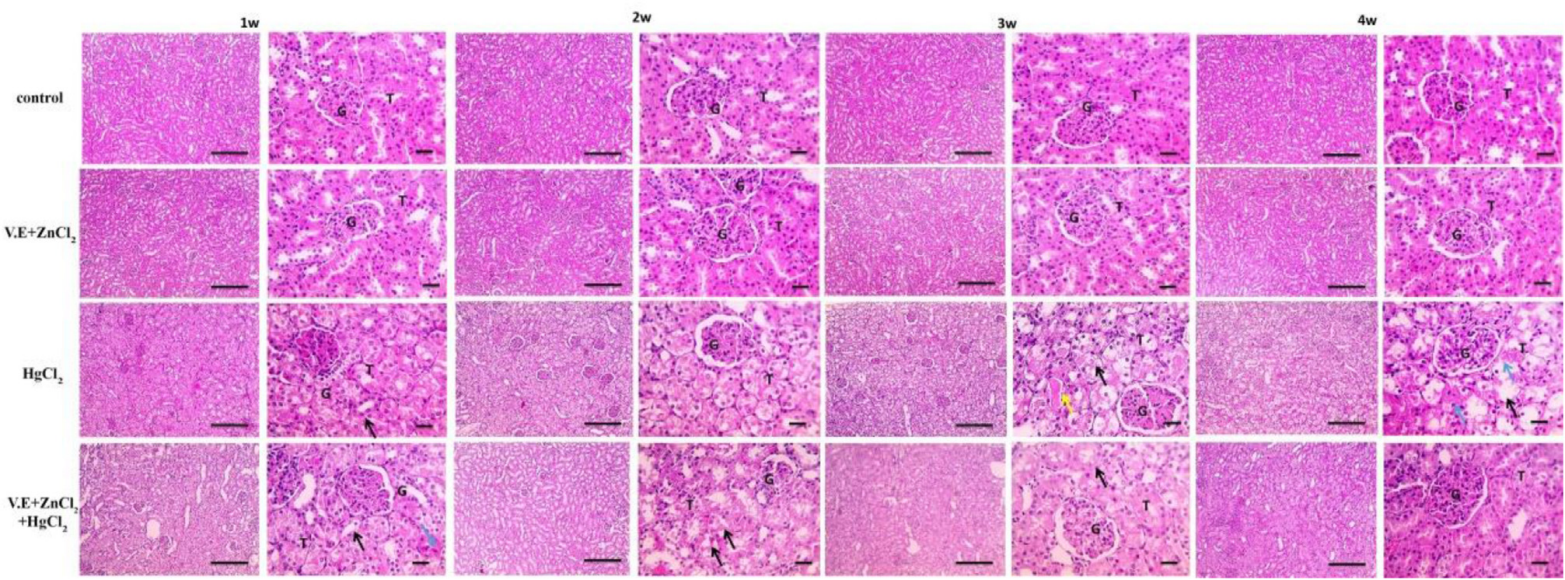
Microscopic pictures of Hematoxylin & Eosin stained kidney sections showing normal glomeruli (G) and tubules (T) in the control group and group receiving zinc chloride and vitamin E. Meanwhile, kidney sections from the mercuric chloride group showed mild tubular epithelial degeneration (black arrows) after one week, slightly increasing after two weeks. Moderate tubular degeneration (black arrows) with tubular cast formation (yellow arrow) appears after three weeks. Severe diffuse tubular degeneration (black arrows) and necrosis (blue arrows) appear after four weeks. After the first week, kidney sections from the mercuric chloride + vitamin E + zinc chloride group showed mild tubular degeneration (black arrows) and necrosis (blue arrows). The severity of congestion gradually decreased from 2 weeks to 4 weeks, where the renal sections retained normal histological pictures of glomeruli (G) and tubules (T). Low magnification X: 100 bar 100, high magnification X: 400 bar 50.

A kidney from the control group displayed a normal histological image with a normal glomerular and tubular architecture. After one week of exposure, treatment with mercuric chloride enhanced mild tubular epithelial degeneration in the kidney. This degradation increased slightly after two weeks, then moderate tubular degeneration with tubular cast formation appeared after three weeks. After four weeks of mercury toxicity, severe diffused tubular degeneration and necrosis occurred. On the other hand, supplementing mercuric chloride-treated rats with vitamin E and zinc chloride showed mild tubular degeneration and necrosis after one week. The severity of congestion gradually decreased and remained normal (Figure 6).

**Figure 7:**
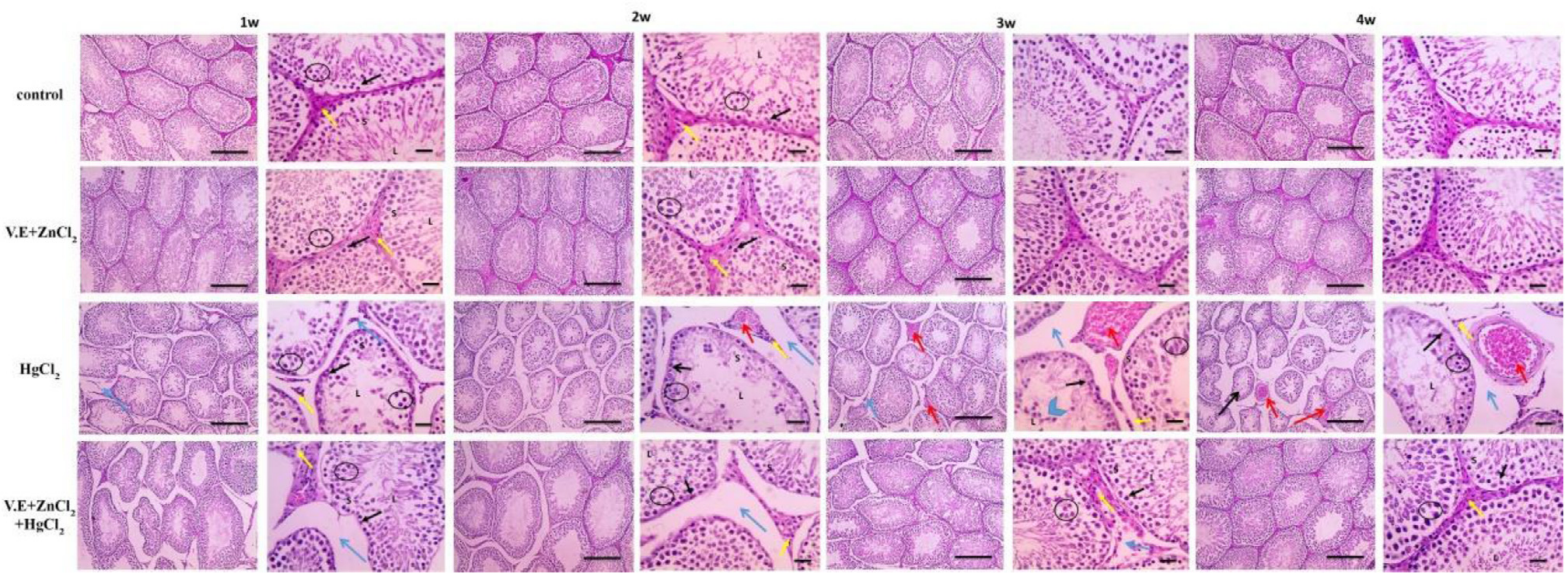
Microscopic pictures of Hematoxylin & Eosin stained testicular sections showed normal regular crossly sectioned seminiferous tubules having a lumen (L) filled with spermatids & spermatozoa and lined with spermatogonia (thin black arrows), several layers of spermatocytes (circle) along with Sertoli cells (S), limited interstitial tissue is seen with normal Leydig cells (yellow arrows) in the control group and group received zinc chloride and vitamin E. Meanwhile, testicular sections from the mercuric chloride group showed few irregular shrunken crossly sectioned seminiferous tubules lined with spermatogonia (thin black arrows) and few layers of spermatocytes (circle) along with Sertoli cells (S), no sperms are seen in lumen (L), increased amount of interstitial tissue (blue arrows), few numbers of small and shrunken Leydig cells (yellow arrows) were observed after the first week. The number of affected tubules and the amount of interstitial tissue increased after the second week, accompanied by congested blood vessels (red arrows). The numbers of affected tubules and congested blood vessels (red arrows), amount of interstitial tissue much more increased after the third and fourth weeks, accompanied by desquamated spermatocytes (blue arrowheads), very few numbers of small and shrunken Leydig cells (yellow arrows). Testicular sections from the mercuric chloride + vitamin E+ zinc chloride group show a few irregular shrunken crossly sectioned seminiferous tubules lined with spermatogonia (thin black arrows) and several layers of spermatocytes (circle) along with Sertoli cells (S), the lumen (L) contains spermatids & spermatozoa and wide interstitial tissue (blue arrows) are seen with increased numbers of normal Leydig cells (yellow arrows) which are observed after one week. The numbers of affected tubules and amount of interstitial tissue decreased after two weeks and much more decreased after 3 & 4 weeks. The testicular sections retained normal histological pictures of seminiferous tubules, a limited amount of interstitial tissue and normal Leydig cells (yellow arrows). Low magnification X: 100 bar 100, high magnification X: 400 bar 50.

**Figure 8:**
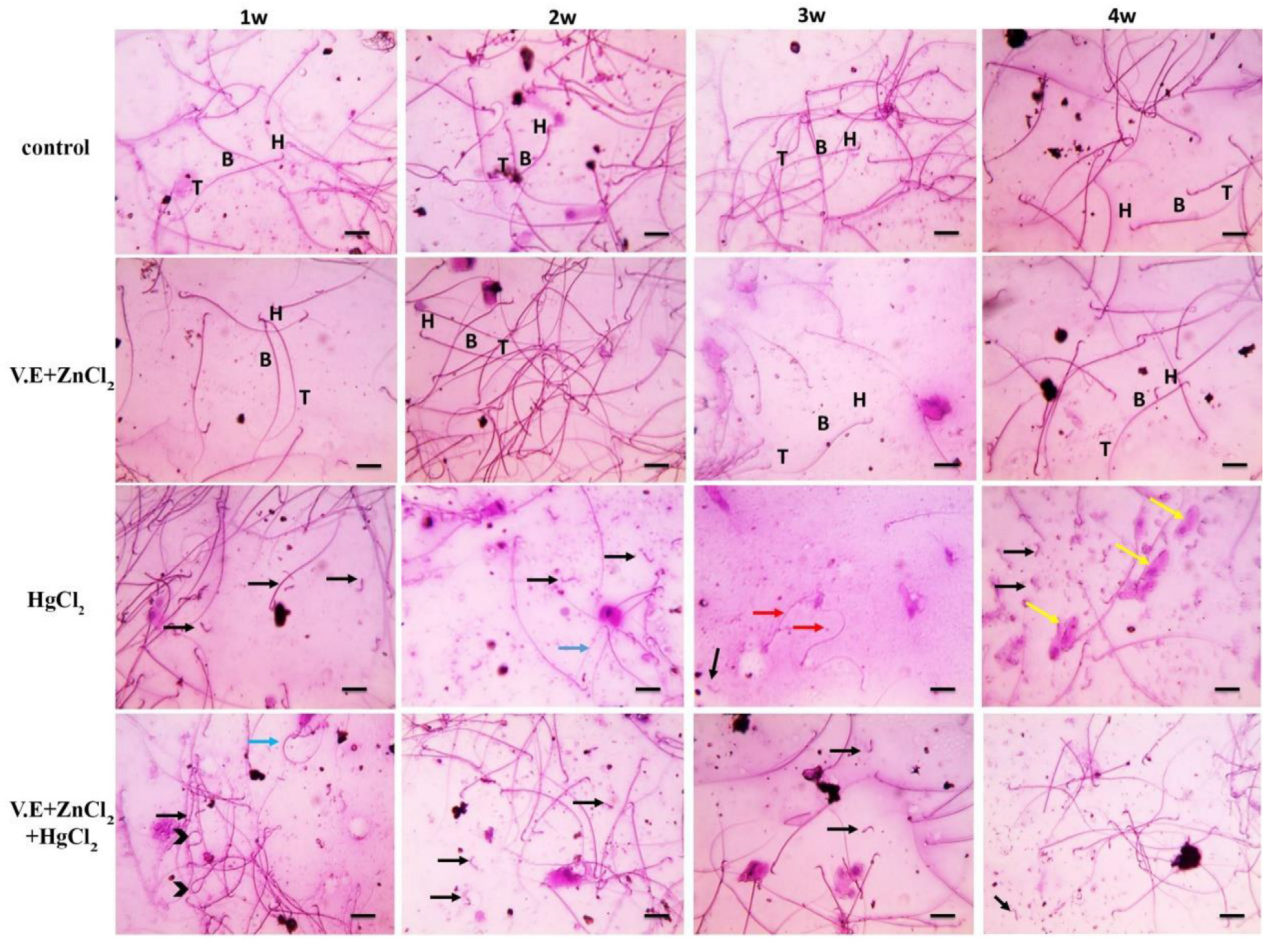
Microscopic pictures of epididymal smear stained with 0.05% aqueous solution of eosin-Y showing normal sperm morphology including head (H), body (B) and tail (T) under the light microscope at 400x magnification in control group and groups received vitamin E + zinc chloride after 1 W, 2W, 3W and 4W. Abnormal sperm morphology in the mercuric chloride group that categorized into mild abnormalities (such as detached head; black arrow) after 1 week, moderate abnormalities (such as detached head; black arrow with the bent middle piece; blue arrow) after 2 weeks, severe abnormalities after 3 weeks such as (detached head; black arrow and dwarf sperms; red arrow); very severe abnormalities after 4 weeks such as (detached head; black arrow with immature sperms; yellow arrows). Epididymal smear from mercuric chloride + vitamin E + zinc chloride group showing moderate abnormalities (such as detached head black arrow, coiled tails arrowheads with bent middle piece blue arrow) after 1 week, mild abnormalities including some detached heads; black arrows after 2 weeks few detached heads; black arrows after 3 weeks and scarce detached head; black arrows after 4 weeks.

Histological architecture of the testis of control group showing normal seminepherous tubules filled with spermatids and spermatozoa. There are several layers of spermatocytes along with normal Sertoli and Leydig cells. Also the epidedimal smear showing normal sperm morphology. Mercuric chloride-induced few irregular shrunken seminiferous tubules lined with spermatogonia and few layers of spermatocytes along with Sertoli cells, no sperms were seen in the lumen, increased amount of interstitial tissue, few numbers of small and shrunken Leydig cells were observed within one week. The number of affected tubules and the amount of interstitial tissue increased after two weeks, accompanied by congested blood vessels. These changes were aggravated by desquamated spermatocytes and very few numbers of small and shrunken Leydig cells after 3 and 4 weeks of treatment. The mercuric chloride group showed abnormal sperm morphology, which was divided into mild abnormalities after one week, moderate abnormalities after two weeks, severe abnormalities after three weeks, such as detached heads, black arrows, and dwarf sperms, red arrows, and very severe abnormalities after four weeks, such as the following: (detached head; black arrow with immature sperms; yellow arrows). On the other hand, supplementation with vitamin E and zinc chloride highly improve these testicular abnormalities (Figures 7 & 8).

**Figure 9:**
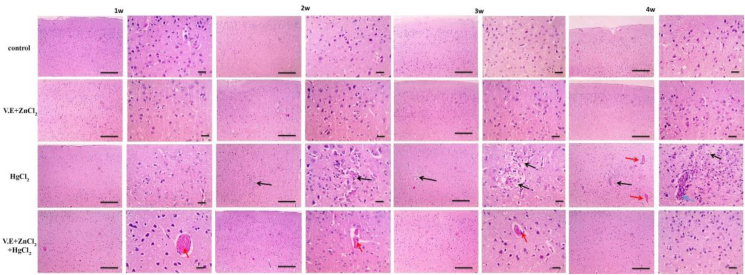
Microscopic pictures of Hematoxylin & Eosin stained cerebral cortical sections showing normal neurons, glial cells and blood vessels in the control group and group that received vitamin E + zinc chloride. No damage is seen in cerebral sections for the mercuric chloride group after one week. A small area of necrosis (black arrows) appears after two weeks, slightly more extensive area of necrosis (black arrows) appears after three weeks. A place of necrosis (black arrows) infiltrated with leukocytes (blue arrows) appears after four weeks, accompanied by congestion of blood vessels (red arrows). Cerebral sections from mercuric chloride + vitamin E + zinc chloride group showing congestion of blood vessels (red arrows) after one week that gradually decreases with time after 2 & 3 weeks and retained normal histological pictures after four weeks. Low magnification X: 100 bar 100 and high magnification X: 400 bar 50.

The cerebral cortical sections showed normal neurons, glial cells and blood vessels in the control group and group that received vitamin E + zinc chloride. Mercuric chloride induced a small area of necrosis after two weeks. Still, a slightly larger size of necrosis after three weeks, the site of necrosis was infiltrated with leukocytes accompanied by congestion of blood vessels by the end of 4 weeks (Figure 9). Administration of vitamin E and zinc chloride with giving rats mercuric chloride-induced congestion of blood vessels after one week, then gradually retained normal by four weeks.

**Figure 10:**
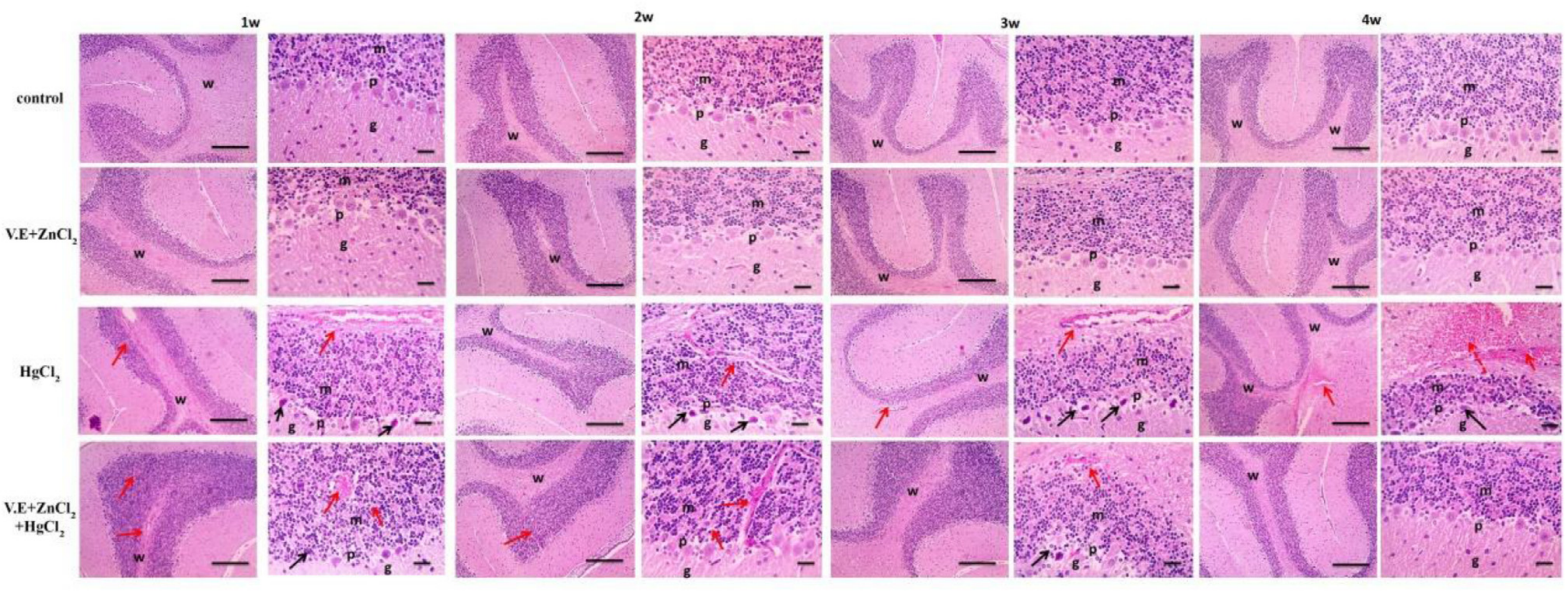
Microscopic pictures of Hematoxylin & Eosin stained cerebellar sections showing normal white matter (w) and grey matter including three layers: granular cell layer (g), molecular cell layer (m) and Purkinje cell layer (p) in the control group and group received vitamin E and zinc chloride. Cerebellar sections from the mercuric chloride group showing shrinkage and degeneration of Purkinje cells (p) characterized by more eosinophilic cytoplasm (black arrows) with congestion of blood vessels (red arrows) after one week. The lesion severity increased with time after two weeks to 4 weeks. Cerebellar sections from the mercuric chloride + vitamin E + zinc chloride group showed few shrunken, degenerated (black arrows) and mild congestion of blood vessels (red arrows) after one week that gradually decreased with time after two weeks & 3 weeks and retained normal histological pictures after four weeks. Low magnification X: 100 bar 100 and high magnification X: 400 bar 50.

In the control group and group that got vitamin E and zinc chloride, cerebellar sections had normal white matter (w) and grey matter, which included three layers: granular cell layer (g), molecular cell layer (m), and Purkinje cell layer (p). Mercury-induced shrinkage and degeneration of Purkinje cells with eosinophilic cytoplasm and congested blood vessels after one week. These disturbances were aggravated by the end of four weeks. Mercuric chloride, vitamin E and zinc chloride induce a few shrunken degenerated and mild congestion of blood vessels after one week, then gradually retained normal by four weeks (Figure 10).

## 4 Discussion

Mercuric chloride induced a significant reduction in body weight (Figure 1A). Bodyweight reduction is an important marker for the disturbance in the rat's general health, and the deterioration of organ weight is important in evaluating organ toxicity (Uzunhisarcikli et al., 2015; Jaiswal et al., 2013). The primary symptom of mercuric chloride toxicity is known to be weight loss, which has been linked to low food consumption of animals (Mahboob et al., 2001). On the other hand, Rao and Sharma (2001) reported that after the administration of HgCl_2_ for 45 days to adult male mice, there was an insignificant increase in the body weight of animals.

Vitamine E and zinc chloride supplementation were found to increase body weight significantly, especially in the third and fourth week of ingestion (Figure 1A). They also improved weight loss induced by mercuric chloride. Pehrson et al. (1991) showed that supplementing animals with vitamin E enhances their weight gain. The increase in fat mass was the primary cause of the weight gain in animals supplemented with vitamin E (Azman et al., 2001). Vitamin E protects against lipolysis and facilitates liposynthesis (Traber, 2013). Vitamin E also stimulates bone growth (Xu et al., 1995) and bone mineral density (Azman et al., 2001). Zinc supplementation has been suggested to boost leptin production and leptin sensitivity (Song et al., 2009; Huang et al., 2004). Leptin is a crucial adipokine that controls energy hemostasis and food intake through central nervous system (Pigeyre et al., 2016; Schwartz et al., 2000). Zinc plays a vital role in regulating appetite and eating habits, through its impact on the synthesis of serotonin and dopamine. Also, zinc has a direct influence on the feeling of satiety and food intake (Johnson, 2001).

Figure 1B-E showed that mercuric chloride induced a significant increase in the relative weight of different organs under investigation. Increased liver weight could be due to the magnifying of the body's adaptive mechanism to combat systemic toxicity (Uzunhisarcikli et al., 2015). Madsen and Maunsbach (1981) demonstrated that the increase in relative kidney weight of rats intoxicated with mercury is due to hyperplasia and/or hypertrophy of proximal tubules. This could be due to elevation in intratubular pressure following obstruction of the tubule by protein casts, the direct effect of mercury on the tubular basement membrane, or its synthesis causing it to lose its normal elasticity and to become more distensible. So, it is clear that the increase in kidney weight is not simply due to the increase in water content of it. The increase in the relative brain body weight ratio after mercuric chloride ingestion suggested the presence of significant tissue inflammation. Increased testicular weight may be attributed to a compensatory change or fluid accumulation in the testis (Boujbiha et al., 2009).

The effects of Mercuric chloride toxicity on the histological structure of different body organs include degeneration and necrosis in hepatic, kidney and cerebral tissues. The cerebellum is shrunk and Purkinje cells have degenerated (Figure 10). Testicular blood vessels were congested and seminiferous tubules and Leydig cells were shrinked (Figure 7). Mercuric chloride-induced spermatocyte immaturity. Our results agreed with those for testis (Prakash et al., 2000; Penna et al., 2009), liver (El-Rhman and Shosha, 2021; Rakib et al., 2021), kidney (Aqeel et al., 2019; Jalili et al., 2020) and brain (Owoeye and Arinola, 2017; Owoeye et al., 2019).

There are findings that link the loss of hepatic architecture with inner mitochondrial membrane depolarization, increase in H_2_O_2_ generation, intensification of lipid peroxidation, and glutathione depletion, stimulated by mercury exposure (Lund et al., 1993; Palmeira and Madeira 1997). Abdel-Moneim (2009) showed that hepatocytes exhibited nucleus heterochromatin scattering, mitochondrial cristolysis, and rough endoplasmic reticulum degranulation when rats were exposed to mercury poisoning. The accumulation of mercuric chloride in the liver causes oxidative stress and liver injury.

Chronic mercuric chloride exposure induced severe tubular degeneration and necrosis in kidney tissue. Mercury can cause oxidative stress by inducing the production of peroxides and superoxide anion radicals. This can lead to membrane lipid peroxidation, protein denaturation, DNA damage, and cellular injury. Furthermore, it has been demonstrated that mercury causes changes in the mitochondrial inner membrane, resulting in an increase in H_2_O_2_ in the mitochondrial electron transport chain and, as a result, a decrease in mitochondrial glutathione levels. These changes in mitochondrial function would result in kidney cell death via apoptosis and/or necrosis (Hazelhoff et al., 2021).

The results of the present study (Figures 5, 6, 7, 8, 9, and 10) showed significant elevations in liver and kidney functions (GPT, GOT, uric acid and creatinine). After treating the animals with Vitamin E and zinc chloride, these elevations retained average values. The marked increase in hepatic enzymes reflects bad liver functions (Al-Attar, 2011). Histopathological studies confirmed also these elevations in liver function as a result of damaging hepatocyte cell membranes due to the toxic effects of mercuric chloride (Uzunhisarcikli et al., 2015). This damage to liver cell membranes could result in the elevation of necrotic and hepatic lesions (Salman et al., 2016). Joshi et al. (2017); Elblehi et al. (2019) and Nabil et al.. (2020) have reported that mercuric chloride causes a significant elevation in AST, ALT, urea and creatinine levels and induction of hepatorenal dysfunction. The higher urea and creatinine levels reflect progressive renal insufficiency in rats treated with mercuric chloride (Oriquat et al., 2012).

The reduction in sperm count indicated the inhibitory effect of mercuric chloride on spermatogenesis (Figure 7). Passing mercury through the blood-testis barrier using drug transporters expressed by Sertoli cells could induce testicular injury (Su et al., 2011). Moreover, table (2) showed that mercuric chloride decreases sperm motility and plasma testosterone concentration. The decline in sperm motility by mercury was thought to be related to inhibiting sperm microtubule structure (Choy et al., 2002). Mercury-induced disorders in spermatogenesis and testosterone hormone (Muthu and Krishnamoorthy, 2012).

In the present research, mercury induced brain cerebral and cerebellar damage. Singlet oxygen can cause damage to cerebral neurons, primarily through the oxidation of essential amino acids. Furthermore, it was discovered that heavy metals inhibit lipid metabolism in animal brains and affect the metabolism of cholesterol, total lipids, and triglycerides in the brain, resulting in an imbalance between lipid synthesis and breakdown. Mercuric chloride is thought to generate reactive oxygen species (ROS) in tissues and increase MDA levels, indicating oxidation of unsaturated fatty acids in the brain and subsequent changes in the anti-oxidative defense system (Omanwar and Fahim, 2015; Teixeira et al., 2018). Takahashi et al. (2017) indicated that mercuric chloride induces an increase in vascular endothelial growth factor expression level in brain tissue that promotes neuronal and blood-brain barrier (BBB) damage by allowing non-selective influx of cytotoxic and inflammatory cells from the blood into brain tissue. Mercuric chloride caused significant neurodegenerative changes in the form of rough endoplasmic reticulum fragmentation, Golgi apparatus ballooning, and nuclear and cytoplasmic degeneration of pyramidal neurons in the rat cerebral cortex (Said et al., 2021).

Due to the ingestion of mercuric chloride in this study, mercury accumulated throughout treatment in the blood and organs, including liver and renal tissues (Figure 3D). Moreover, it induced hepatic and renal apoptotic DNA fragmentation (Figure 4A, B). This accumulation of mercury in the tissues leads to disrupt many organs functions such as liver and kidney through both vasoconstriction and direct cytotoxicity on podocyte cells (Barregard et al., 2010). The mechanism of action of mercuric chloride on most of the body cells is through binding to sulfur. It replaces the hydrogen ion in the body's sulphydryl groups, leading to disruption in cells (Cappelletti et al., 2019). It also reacts with phosphoryl carboxyl and amide, causing alterations in cell membranes, enzymes, transport mechanisms, structural proteins and nucleic acid synthesis (Morakinyo et al., 2012). On the other hand, mercuric chloride is found to promote oxidative stress and apoptosis by generating ROS, oxidizing glutathione and damaging DNA in PC12 cells (Hossain et al., 2021). Mercury elevates the production of intracellular ROS (Durak et al., 2010). Malondialdehyde, the last lipid peroxidation product and an important oxidative stress indicator, increased significantly (*p < 0.05*) at different periods of mercuric chloride ingestion. Su et al. (2008) showed that MDA concentration elucidates the percentage of damaged cells and tissues. Agarwal et al. (2010), Aslanturk et al. (2014), and Kalender et al. (2013) reported increased levels of MDA in various tissues and plasma in response to mercuric chloride toxicity. It was shown that high MDA levels are an indicator of liver tissue damage (Uzunhisarcikli et al., 2015).

It was demonstrated that the cellular antioxidant defense system (e.g., SOD and CAT) controls the potentially harmful effects of free radicals generated by mercuric chloride (Faix et al., 2003). The superoxide dismutase enzyme catalyzes the dismutation of superoxide radicals to hydrogen peroxide and molecular oxygen (Boujbiha et al., 2009). Catalase converts H_2_O_2_ to H_2_O and oxygen, protecting cells from oxidative damage (Renugadevi and Prabu, 2010). This study demonstrated the ability of mercuric chloride to induce oxidative stress by increased lipid peroxidation as shown in decreasing plasma SOD and CAT activities and increasing plasma MDA. This may be correlated with decreased SOD and CAT activities in mercuric chloride-treated rats' liver and kidney tissues (El-Sawi et al., 2017).

Mercuric chloride promotes alteration in the hemopoietic system (El-Demerdash et al., 2004). This study showed that mercuric chloride-induced significant elevation in WBCs count. Mercuric chloride increases abnormal leukocytosis, which may damage the organ's tissues through its immune response action (Mahour and Saxena, 2009). The histopathological alterations induced by mercury could provoke the elevation in WBCs count (Kalender et al., 2010). Results indicated that mercuric chloride reduced red blood cell count and hemoglobin concentration (Figure 2A, B). Accumulation of mercury in RBCs leads to alterations in the erythrocyte physiology due to changes in the oxygen-binding capacity of hemoglobin and a decline in band 3-mediated ion exchange (Ahmad and Mahmood, 2019; Piscopo et al., 2020). Mercury was found to induce hemolytic and aplastic anemia as mercury competes with iron for binding to hemoglobin which can result in impaired hemoglobin formation (Saljooghi and Delavar-mendi., 2013). The present finding aligned with those reported by Chemelo et al. (2021) and Mohamed et al. (2019). They observed a decrease in RBCs count and Hb concentration and damage to the peripheral blood in response to mercury toxicity.

The current results showed that vitamin E and zinc chloride supplementation improved different lesions induced by mercuric chloride. Vitamin E is an antioxidant that prevents lipid peroxidation and the formation of free radicals and maintains ascorbic acid and glutathione levels in damaged tissues (Rao and Patil, 2000; Oda and El-Maddawy, 2012). It inhibits lipid peroxidation by scavenging free radicals (Uzunhisarcikli and Kalender, 2011). Also, it impaired mercury absorption (Rao and Sharma, 2001). Moreover, vitamin E can also inhibit the conversion of –SH to SS groups; thus, it maintains the protein functions of tissues (Basu and Dickerson, 1996; Rao and Patil, 2000). Vitamin E decreases the storage and toxicity of reactive oxygen species (Aslam et al., 2010). Vitamin E destroys hydroperoxides and thus protects cells and organelles membranes from peroxidative damage (Gupta et al., 2005). Vitamin E minimizes nephrotoxicity (Aslanturk et al., 2014), testicular toxicity (Kalender et al., 2013), and improves hematological, hepatic (Alhusaini et al., 2021) and lung toxicity (Celikoglu et al., 2015).

Vitamin E could have a role in forming selenium mercury complexes in tissues, decreasing mercury toxicity, primarily by reducing mercury absorption through the elementary canal (Uzunhisarcikli et al., 2015). Also, selenium antagonizes mercury toxicity in the brain of chickens by promoting the signaling pathways BDNF/TrKB/PI3K/AKT and suppressing NF-kappaB (Li et al., 2022). It reduces the liver damage caused by mercuric chloride by controlling mitochondrial dynamics to prevent the interaction between energy metabolism dysfunction and NF–B/NLRP3 inflammasome-mediated inflammation (Gao et al., 2021). Selenium can counteract mercuric chloride-induced oxidative stress and inflammation in the central immunological organs of chickens through a new mechanism involving the miR-135b/183-FOXO1/TXNIP/NLRP3 inflammasome axis (Gao et al., 2022) Muthu and Krishnamoorthy (2012) showed that vitamin E could reduce testicular injury by normalizing the antioxidant activity in the testis and declining malondialdehyde and inflammatory cytokines (Fadda et al., 2020). On the other hand, it was found that administration of vitamin E reduces HgCl_2_-oxidative damage and prevents apoptotic and inflammatory markers in the liver, thus improving liver morphology (Alhusaini et al., 2021).

Zinc is essential in activating many enzymes (Sandstead and Au, 2007; Shah, 2011). Zinc eliminated mercury from rat liver and kidneys (Oliveira et al., 2014). Zinc enhances the synthesis of scavengers like glutathione and metallothionein in tissues, and it has a definite role in the excretion and elimination of mercury. It was reported that zinc significantly reduced hepatic mercury levels and increased renal mercury levels (Peixoto et al., 2003; 2007; 2008; Franciscato et al., 2011).

Vitamin E and zinc chloride ingestion reduce renal excretion of mercury and decline its concentration in the liver, total blood and kidneys in rats treated with mercuric chloride. This gives the impression that vitamin E and/or zinc chloride may interact with mercury or affect/s its metabolism, or enhance/s its excretion through another way; e.g. gastrointestinal tract, sweat, or divert/s its storage to another body organ/s, and this needs further study.

## 5 Conclusions

Mercuric chloride's toxicity impacts normal architecture of rat's organs such as the liver, kidney, testis and brain. It elevated the white blood cell count that infiltrated into the tissues and aggravated anemia by reducing RBCs and hemoglobin. It extensively induced oxidative stress and lipid peroxidation due to mercury accumulation in the liver, kidney and blood cells. It enhances apoptotic DNA fragmentation in liver and kidney inflamed tissues leading to changes in their functions. Mercury reduces body weights accompanied by severe depression in sperm count, sperm motility and testosterone levels and enhances abnormal sperm morphology. Supplementation with vitamin E and Zinc chloride improves many alterations induced by mercuric chloride although further studies should be done to determine fate for mercury.

## Declarations

### Author contribution statement

Mohamed Gaber Shalan: Conceived and designed the experiments; Performed the experiments; Analyzed and interpreted the data; Contributed reagents, materials, analysis tools or data; Wrote the paper.

### Funding statement

This research did not receive any specific grant from funding agencies in the public, commercial, or not-for-profit sectors.

### Data availability statement

Data will be made available on request.

### Declaration of interest's statement

The authors declare no conflict of interest.

### Additional information

No additional information is available for this paper.

## Figures and Tables

**Figure (4A) Not adjusted:**